# Annual Research Review: Early conduct problems – precursors, outcomes, and etiology

**DOI:** 10.1111/jcpp.70031

**Published:** 2025-08-14

**Authors:** Luke W. Hyde, Christopher J. Trentacosta, Jessica L. Bezek

**Affiliations:** ^1^ Department of Psychology University of Michigan Ann Arbor MI USA; ^2^ Department of Psychology Wayne State University Detroit MI USA

**Keywords:** Conduct problems, parenting, coercive cycles, developmental psychopathology, genetics, neuroscience

## Abstract

During the toddler and preschool period, nearly all children engage in some level of aggression, defiance, stealing, and temper tantrums. While the frequency and intensity of these behaviors tends to decrease across early childhood, a subset of children engage in these conduct problem behaviors at a higher intensity early in life and/or do not desist from these behaviors. Instead, these behaviors escalate across childhood and adolescence into serious forms of antisocial behavior (e.g., aggression, rule breaking). Given the negative impacts of these behaviors on children engaging in them, victims, and society, childhood conduct problems are a major public health concern. Here, we provide an updated review of the research identifying the trajectory of conduct problems; risk factors for their emergence, persistence, and escalation; and mechanisms through which risk impacts behavior, using a biopsychosocial and ecological lens. We describe how child and parent attributes contribute to a coercive dyadic cycle that leads to escalating problem behaviors, and how the broader context undermines these proximal relationships to increase risk for conduct problems. Next, we consider the role that genetics plays in these processes and describe how gene–environment interplay shapes early conduct problems. Further, we describe the ways in which these environmental and genetic risk processes impact brain development to increase risk for conduct problems. Finally, we discuss recent approaches to prevent and treat conduct problems and discuss research needs to better support our understanding of the development, prevention, and treatment of early conduct problems.

## Introduction

Temper outbursts, defiance, aggression, intentionally breaking things, and taking things that are not yours are relatively normative behaviors in the first years of life. Yet, these conduct problems are also hallmarks of the antisocial behavior characterized in later developmental periods as the disruptive behavior disorders of Conduct Disorder (CD) and Oppositional Defiant Disorder (ODD) in the DSM and ICD (American Psychiatric Association, 2013; World Health Organization, 2019). Though normative early in life, when these behaviors persist and escalate into serious antisocial behavior, they disrupt the developmental trajectories of youth engaged in these behaviors, along with creating major impacts on victims, families, and society. Youth who engage in serious antisocial behavior in adolescence cost society 10× more in service utilization than other youth (Scott, Knapp, Henderson, & Maughan, 2001). For victims, the costs of serious antisocial behavior can be immeasurable, including physical and psychological injury (Miller, Cohen, & Rossman, 1993), as well as the broader impacts of violence and crime on communities (Sharkey, 2018). Moreover, in addition to antisocial behavior, early conduct problems are also associated with a higher risk for a broad array of later mental and physical health problems (Kretschmer et al., 2014; Moffitt, 2018), which each have their own impacts. Thus, conduct problems are a major public health problem. However, how do behaviors that are relatively normative early in life persist, become less normative and more problematic, and transition to serious antisocial behavior and violence? This review underscores that the first years of life are a critical juncture in the initiation, persistence, and escalation of conduct problems that transition to serious antisocial behaviors and, thus, are an essential developmental period for recognizing, preventing, and addressing conduct problems.

**Figure 1:**
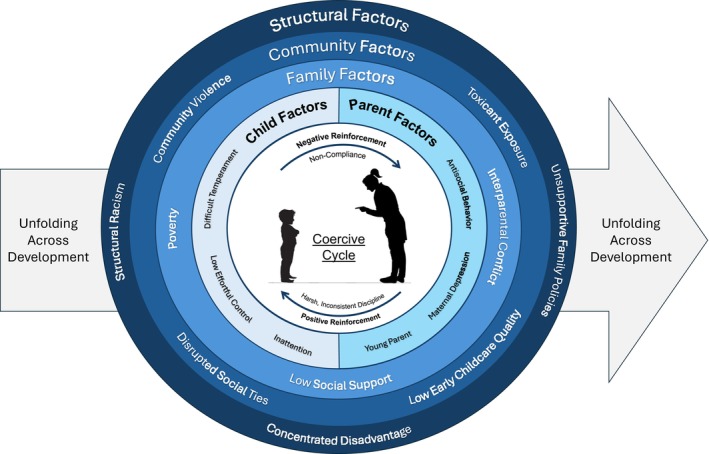
Multi‐level risk pathways for conduct disorder. Risk for conduct problems emerges most proximally at the parent–child level through coercive cycles. However, risk for these cycles emerges from a complex interaction between multiple levels of risk that is filtered through more proximal contexts. For example, broader systems and policies impact more proximal contexts like childcare and parent stress, which, in turn, directly impacts child–caregiver interactions. Parent and child risk are also the result of histories and unfold from conception through childhood at multiple interacting levels. This figure highlights just a few examples of risk factors at each level

This review summarizes the literature on the development of conduct problems early in life (i.e., from the toddler into school age years) and the causes of their emergence, persistence, and escalation. We describe what is currently known about factors that predict early emerging conduct problems, factors that may exacerbate or inhibit the persistence and escalation of these behaviors into the school years and beyond, and the science of treating and preventing conduct problems. Much of the research in this area has focused on child–caregiver interactions that escalate over time leading to more severe and stable behavior. These interactions are shaped by aspects of the child and caregiver as well as multiple layers of the social context. Thus, we review risk factors the child, the parent, and their interactions. Then, we zoom out to describe the social contexts that shape these parent–child relationships. Next, we describe how genetic and environmental factors interact to increase risk for conduct problems and their escalation as well as the neural mechanisms that underlie these problematic trajectories. We end by highlighting cutting‐edge prevention and intervention strategies and research and treatment needs in the field. Our review is guided by Figure 1, which highlights the multi‐level nature of the ecology of early conduct problems.

## Early conduct problems: Definitions, development, and problematic persistence

Early conduct ‘problems’ are typically outgrowths of relatively common and normative behaviors that occur during the early years of life such as temper tantrums and hitting (Lorber, Del Vecchio, & Slep, 2018). Anger, aggression, and impulsive physical behaviors increase during the ‘terrible twos’ followed by a normative decline from 3 to 4 years (Alink et al., 2006). During the first 3 years of life, children's brains develop rapidly, and they have newfound mobility and desires. However, infants and younger toddlers lack neurobehavioral control and experience with the world to inhibit potentially dangerous and problematic behaviors, which presents a new challenge for parents. Fortunately, early problem behaviors decline for most young children as continued brain development leads to better self‐regulation of emotions and behaviors (Rothbart, Sheese, Rueda, & Posner, 2011) and as caregivers promote compliance and socialize their children to appreciate the consequences of their actions and to understand and regulate their emotions (Johnson, Hawes, Eisenberg, Kohlhoff, & Dudeney, 2017; Kochanska, Boldt, & Goffin, 2019; Shaw & Bell, 1993).

Even though conduct problems are normative in the first few years of life, it is possible to identify a subset of children for whom conduct problems may become chronic and persistent (though note that none of the work we review should be taken to suggest that any *specific* child with conduct problems is *destined* to show serious or chronic antisocial behavior). Seminal work by Moffit (2018) described an early (before age 10) versus late starter (typically during adolescence) model of antisocial behavior. A small but important group of ‘early starting’ youth (~4%) showed consistent antisocial behavior from before age 10 that persisted well into adulthood (Moffitt, 2018). Seminal work earlier in life showed that half of the ‘hard‐to‐manage’ preschoolers at age three (i.e., those exhibiting greater noncompliance, aggression, inattention, hyperactivity) continued to exhibit adjustment difficulties by age six (Campbell, Ewing, Breaux, & Szumowski, 1986). Several subsequent longitudinal studies have supported the notion that children at‐risk for school‐age conduct problems can be identified based on the presence of elevated conduct problems during the first few years of life. For example, in a longitudinal study of children with enrichment for early conduct problems, 4% of the sample had stably elevated conduct problems from age 3 to age 10 (Olson, Choe, & Sameroff, 2017). Similarly, using group based trajectory modeling, Shaw, Gilliom, Ingoldsby, and Nagin (2003) found that a small portion (6%) of an at‐risk sample of boys from low‐income families demonstrated consistently high levels of conduct problems from age 2 to age 8. Multiple other longitudinal studies provide evidence for small subsets of young children with trajectories of elevated early conduct problems that persist for years into late childhood and adolescence (e.g., Perry, Calkins, Dollar, Keane, & Shanahan, 2018). Importantly, across these longitudinal studies, a comparatively larger subset of the children (between 16% and 38%) showed relatively high initial levels of conduct problems that decreased over time.

Given that a small subset of children display early conduct problems that become stable and chronic, whereas a larger subset of children display early conduct problems that desist over time, it is reasonable to wonder ‘when to worry’ about early problems (Wakschlag et al., 2019). As Wakschlag et al. note, several factors influence the extent to which early problems are concerning, including the quality of the behavior, the extent to which the behavior is developmentally expectable within a given context, the regularity of occurrence (ideally assessed as concretely as possible), and the specific spectrum of behaviors within narrow‐band components of conduct problems (e.g., temper loss). As one example, toddler misbehaviors like tantrums may become more concerning if they occur very frequently (e.g., daily) or if they are qualitatively distinct (e.g., destructive tantrums). These factors underscore the importance of evidence‐based assessment of early childhood conduct problems (Wakschlag et al., 2008; Wakschlag et al., 2014).

## Child, caregiver, and dyadic processes of risk for conduct problems

### Child risk

Several child factors increase risk for the early emergence of more severe conduct problem behaviors (e.g., early emerging temperament factors), while other child factors may indicate risk for persistence (e.g., gender) and/or a different etiology that may be more severe and persistent (e.g., callous‐unemotional traits).

#### Birth outcomes

Children can begin life with increased risk for conduct problems. Children born preterm, extremely low birth weight, or with delivery complications often have more externalizing problems (Beck & Shaw, 2005; Bhutta, Cleves, Casey, Cradock, & Anand, 2002; Mathewson et al., 2017), although these associations are not always consistent (e.g., Cassiano, Gaspardo, & Linhares, 2016). A thorough summary is beyond the scope of this review, but numerous prenatal factors set the stage for harmful birth outcomes; for example, lead exposure is linked to preterm birth (Lanphear, Navas‐Acien, & Bellinger, 2024).

#### Biological sex and gender

Early in life, there are relatively few sex differences in problem behaviors, but by age four, girls tend to have fewer conduct problems than boys. These sex differences are due, in part, to socialization of girls toward internalizing and away from externalizing problems (Keenan & Shaw, 1997), which aligns with gender‐related norms for expressing internalizing (e.g., sadness) versus externalizing (e.g., anger) emotions (Chaplin, 2015). Factors that increase the risk of conduct problems may differ for boys and girls. For example, in a longitudinal study across early childhood, inattention predicted membership in a chronic profile for both boys and girls, but emotion regulation and socioeconomic status were unique predictors for girls versus boys, respectively (Hill, Degnan, Calkins, & Keane, 2006). Increased prenatal testosterone exposure could be another factor, with increased testosterone exposure linked to traits including low empathy, low effortful control, and high sensation seeking, as well as aggression and rough‐and‐tumble play behaviors that are more common among males (Martel, 2013). Thus, early differences related to biological sex may increase risk for conduct problems, but socialization of boys versus girls during the preschool years creates additional risk for boys toward conduct problems.

#### Temperament

There are several temperament characteristics that place children at greater risk for early emerging (and more persistent) conduct problems. A large body of literature has focused on ‘difficult temperament’ (Bates, 1980), which is characterized by a combination of negative emotionality and difficulty with self‐regulation (e.g., Kochanska & Kim, 2013). Indicators of difficult temperament have been linked to conduct problems (Frick & Morris, 2004), including early‐onset persistent conduct problems (Barker & Maughan, 2009). Though most of the research linking difficult temperament with conduct problems has started at age two or later, more intense and frequent crying/distress, low soothability, and irritability are among the earliest markers of difficult temperament from the first days of life that make infants more challenging to manage (Barr et al., 2014) and increase risk for early conduct problems (Finlay‐Jones et al., 2024).

In addition to difficult temperament, specific temperament facets, including anger, impulsivity, and effortful control (the capacity to initiate a subdominant response while inhibiting a dominant response), are each linked to conduct problems (Eisenberg et al., 2001). Notably, hallmark behaviors associated with poor effortful control and impulsivity are well represented in the symptom profile for Attention‐Deficit Hyperactivity Disorder (ADHD). ADHD is highly comorbid at later ages with Conduct Disorder, with as many as 90% of youth with Conduct Disorder having an ADHD diagnosis (Danielson et al., 2024). Thus, many children with conduct problems will also have high levels of ADHD symptoms. Beyond attention difficulties, reward dominance and punishment insensitivity have also been linked to childhood conduct problems and may disrupt parental attempts at discipline (Dadds & Salmon, 2003). Finally, fearlessness is another specific facet of temperament that may make young children more impulsive and difficult to manage and has been linked to conduct problems (Fanti, Mavrommatis, Colins, & Andershed, 2023; Shaw et al., 2003).

#### Callous‐unemotional traits

Callous‐unemotional (CU) traits, which include low guilt, prosocial emotions, and concern for others, capture aspects of temperament described above including fearlessness (Waller et al., 2016). Much of the research on CU traits has focused on adolescence or later childhood. Youth with antisocial behavior and CU traits appear to have distinct risk profiles compared to youth with only antisocial behavior (Frick, Ray, Thornton, & Kahn, 2014), with CU traits marking a form of antisocial behavior that has a different etiology, is more highly heritable, has different neural correlates and may be more difficult to address with existing treatments (Hyde & Dotterer, 2022).

In the last decade, emerging research has found that CU behaviors, as early as age 3, predict later CU traits, along with more stable and chronic trajectories of conduct problems (Waller et al., 2016). For example, in a study of low‐income families, most youth were decreasing in their conduct problems from age 2 to 4, but youth high on CU behaviors showed an increasing trajectory (Hyde et al., 2013). Thus, the presence of CU behaviors may identify young children with more serious conduct problems who may have different treatment needs.

### Parent risk

Parenting is one of the most studied factors related to the ecology of early conduct problems. Harsh and rejecting parenting, defined by hostile, critical, and punitive responses to children (Pardini, Waller, & Hawes, 2015), is robustly linked to early‐emerging conduct problems (Campbell, 1995; Shaw et al., 2003). Harsh parenting is theorized to pose risk for child conduct problems for multiple reasons, including that it models aggression and undermines the parent–child bond in ways that make other parental socialization efforts ineffective. Beyond harsh parenting, parenting that is lax and/or inconsistent has also been robustly linked to conduct problems (Campbell, 1995). Lack of firm, consistent boundaries provides inconsistent messages about behavior for children and can contribute to dyadic processes that inadvertently reinforce negative child behavior.

In addition to harsh and inconsistent parenting, parenting that is low in warmth and positive reinforcement has also been linked to conduct problems and later severe antisocial behavior (Hoeve et al., 2009). Harsh parenting and low parental warmth are typically only modestly negatively correlated and each predict increases in conduct problems over time, both over and above the stability of conduct problems and beyond child effects (Pinquart, 2017). However, though both are important in the development of conduct problems, harsh parenting appears to have somewhat larger effect sizes than aspects of (low) warmth and positive reinforcement (Hoeve et al., 2009; Pinquart, 2017). Interestingly, low warmth may be particularly important for the development of CU traits (Waller, Hyde, Klump, & Burt, 2018).

Seminal work in the development of prosocial behavior suggests that parenting is key in socializing children to exhibit fewer problem behaviors and more prosocial behavior over time (Eisenberg, Cumberland, & Spinrad, 1998; Kochanska, 1993). According to early social control theories (Hirschi, 1969), problem behaviors are diminished via bonds to society, particularly attachment to parents and other people who introduce and reinforce the value of prosocial behavior. Secure parent–child attachment promotes effective socialization early in life by interrupting negative cascades (e.g., difficult child temperament, negative parental control) and increasing children's acceptance of parental socialization/rule‐setting across development (Kochanska et al., 2019). A strong parent–child bond leads to a child who is more receptive to parental guidance and socialization, whereas insecure or disorganized attachment relationships are robustly associated with conduct problems (Fearon, Bakermans‐Kranenburg, Van IJzendoorn, Lapsley, & Roisman, 2010). Specific parental behaviors associated with positive socialization include parental modeling (e.g., helping others), emotion socialization (e.g., discussing emotions, fostering understanding of others' emotions), and demonstrating inductive reasoning (i.e., explaining rules and consequences to children; Hastings, Utendale, & Sullivan, 2007). Importantly, there are individual differences in children's openness to parental influence (Tremblay, 2005), with some children more easily socialized to reduce disruptive behavior in the face of negative feedback from parents and authority figures and others more resistant to this socialization and, thus, requiring stronger parental shaping of positive behavior and other environmental modifications.

### Parent–child interactions

Seminal developmental theories that address the emergence and persistence of conduct problems emphasize the intersection of child (e.g., difficult temperament) and parenting (e.g., harshness) risk as they unfold over time during parent–child dyadic interactions. The interplay of child and family risk factors is particularly relevant during moment‐to‐moment interactions between children and their parents because they can create patterns that increase risk for longer‐term conduct problems. Social learning theory models (Dishion & Patterson, 2006; Scaramella & Leve, 2004), along with theories that integrate the social learning perspective with attachment models (Shaw & Bell, 1993), describe the unfolding of early conduct problems as they emerge from parent–child interactions across the first 5 years of life. In these transactional models, coercive cycles emerge between children and parents that persist and escalate across developmental periods and eventually spread to other settings (e.g., other caregivers, teachers, peers; Dishion & Patterson, 2006).

**Figure 2:**
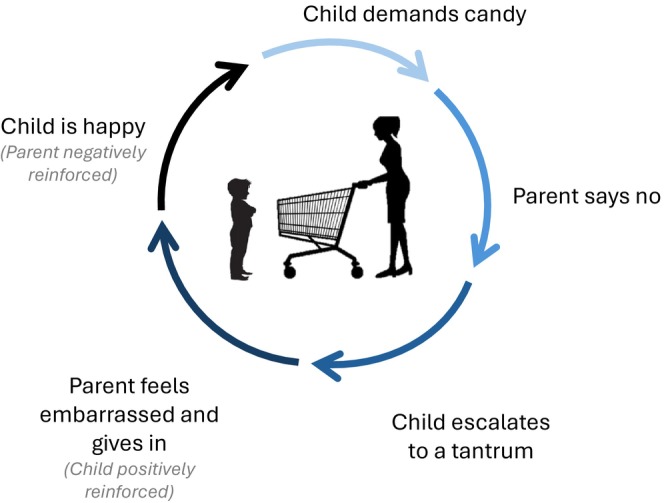
An illustrative microexample of the coercive cycle. Summary of the illustrative microexample: During grocery shopping, a child demands candy. At first, the parent holds firm that the child cannot have candy. However, the child escalates to a public temper tantrum and, feeling embarrassed and frustrated, the parent gives in. The child is thus inadvertently rewarded for this behavior. The parent has also been reinforced (negative reinforcement) because the child's aversive behavior has stopped. The parent may later become angry and yell or engage in other harsh discipline, but the aversive behavior is likely to increase in frequency and/or intensity in future episodes, at least in part due to the fact that the child has already received immediate positive reinforcement and the punishment came later and was not contingent (and was received by a child who may be less sensitive to punishment). In subsequent trips to the grocery store, the parent is more likely to give in quickly, or, when they do try to stand their ground, the child will escalate even further to gain the candy

In the first 2 years of life, children who are more temperamentally difficult challenge parents' own emotion regulation, which can lead to harsher and more inconsistent parenting (Scaramella & Leve, 2004). Moreover, babies who are harder to soothe and more irritable can tax parents' limited emotional resources, leading to less warmth and engagement. Of course, this process is dyadic and shaped by the developing attachment relationship (Calkins & Leerkes, 2004), and thus parents' responses to their baby's distress may also undermine their child's emerging ability to emotionally self‐regulate, which in turn will make the child more difficult to soothe. As children begin to toddle and walk, their newfound physical capabilities emerge as they are still learning to regulate their emotions. This period is a challenge for all parents but can be particularly challenging for a child with greater emotion dysregulation and for parents who may also be struggling with their own emotion regulation difficulties. Interactions can become entrenched in coercive cycles in which parents give in to their child's escalating behavior that, in turn, reinforces this behavior. As parents become frustrated with escalating behavior, they may intermittently respond, often in harsh ways, which might be effective in the short term, but ineffective over time because the child learns to ignore or escalate. This pattern becomes one of both harsh and inconsistent parental responses. Over time, children, particularly those who are more fearless and less sensitive to punishment, learn that escalating their behavior is effective at achieving their goals. In parallel, parents learn that giving in often stops aversive behavior in the short term, which yields a negative reinforcement cycle that teaches parents to give in to negative child behavior. Moreover, these interactions limit opportunities for positive and rewarding interactions, which undermines the parent–child bond and attachment, leading to less incentive for either member of the dyad to engage in positive ways. To illustrate this cycle, see Figure 2. It is important to note that these patterns of behavior are learned implicitly and are not necessarily volitional either by the parent or the child. ‘Decisions’ to engage in these behaviors (often when emotions are high ‘in the heat of the moment’) are based on prior history (e.g., which behaviors have been reinforced). As a result, these behaviors can become entrenched in the parent–child dyad even if they do not ‘want’ to engage in them; an important concept to consider for treatment providers and parents.

A wealth of literature has supported the coercive cycle and other relational processes (e.g., peer deviancy training) that contribute to early conduct problems and later antisocial behavior (Dishion & Patterson, 2006), including research informed by dynamic systems perspectives that elucidate how facets of the dyad such as rigidity contribute to entrenched patterns of problem behavior (Granic & Patterson, 2006; Lunkenheimer, Olson, Hollenstein, Sameroff, & Winter, 2011). Recent research has also highlighted several factors that may modulate these cycles, including CU traits and attachment. For example, Kochanska, Kim, Boldt, and Yoon (2013) found that among young children with elevated CU traits, higher mother–child mutually responsive orientation and father–child shared positive affect predicted fewer future conduct problems, but this association was not found among young children with relatively lower CU traits. This finding aligns with the ‘goodness of fit’ perspective (Chess & Thomas, 1991), which suggests that aspects of the environment, including the caregiving context, may be especially compatible for some children, but less so for others, based on their temperament characteristics. In another example of moderation of the coercive cycle, toddlers' anger proneness was related to parents' power assertive discipline and children's subsequent conduct problems, but only among insecurely attached parent–child dyads (Kochanska & Kim, 2012). The coercive cycle can also be exacerbated or mitigated by the parent's and child's internal working models, which are mental representations of each other that emerge in the context of the early attachment relationship (Bretherton & Munholland, 2008). For example, the pathway from infant difficult temperament to early childhood disruptive behavior via maternal power assertive discipline during toddlerhood was only present when mothers and young children had negative internal working models of each other (Kochanska & An, 2024). This finding indicates that more positive mental representations in the mother–child dyad can help stave off cycles leading to early conduct problems.

## The broader context

Though seminal work on parenting has helped inform effective interventions, parenting itself is a more proximal mediator of broader socio‐contextual risk (Taraban & Shaw, 2018). That is, coercive cycles are not merely the product of challenging children, harsh parents and their underlying relationship, but the product of systems that increase risk exposures for parents and children across developmental periods and undermine the abilities of parents to provide effective parenting.

### Prenatal risk factors

Contextual risk factors exert their influence even before birth. For example, meta‐analytic evidence indicates that prenatal distress is associated with externalizing problems even after adjusting for postnatal distress (Tung et al., 2024). Other meta‐analytic evidence indicates that smoking and alcohol consumption during pregnancy are associated with conduct problems (Ruisch, Dietrich, Glennon, Buitelaar, & Hoekstra, 2018). However, as we discuss below, genetic confounding in these designs is a key limitation, even in commonly used genetically informed designs, because they cannot disentangle the impact of birth mother genetics from the prenatal environment (Rice, Langley, Woodford, Smith, & Thapar, 2018). Other exposures during pregnancy are via the broader physical environment. For example, lead exposure, which can stem from prenatal as well as postnatal influences, has been linked to conduct problems (Marcus, Fulton, & Clarke, 2010).

### Demographic, family context, and neighborhood risk

Some of the most consistent predictors of child conduct problems and later youth antisocial behavior are indicators of disadvantage and low income. Seminal work on the family stress model posits that low income and financial insecurity impact children by stressing parents, which, in turn, undermines positive family relationships (Conger & Donnellan, 2007; Shaw & Shelleby, 2014). From this perspective, part of what leads to coercive cycles is that parents do not have the time or emotional resources to be more consistent and engaged. Several other specific sociodemographic, family, and parental risk factors have also been linked to child conduct problems and later antisocial behaviors, including maternal depression, parental antisocial behavior, interparental conflict, single parent and teen parent status, daily hassles, and low social support (for a review, see Hinshaw & Lee, 2003). Collectively, these factors undermine parents' ability to be warmly engaged and provide firm, consistent limits. For example, a single teen parent who is suffering from depression and juggling multiple low‐wage jobs may struggle with the time and emotional resources to consistently engage in positive ways with their young child, particularly one that may have a more difficult temperament. Importantly, these factors may co‐occur, leading to ‘cumulative risk’ (Sameroff & Seifer, 1995). As an example of this pathway, in a large sample of lower‐income families, cumulative risk at age 2 predicted youth conduct problems at age 4 via lower levels of nurturant and involved parenting at age 3 (Trentacosta et al., 2008).

Similarly, living in a neighborhood with fewer resources can lead to additional stress to parents via both greater challenges in obtaining goods (e.g., lack of nearby grocery stores) and employment (lack of transportation to a job), as well as via exposure to community violence (Leventhal & Brooks‐Gunn, 2000), which may undermine children's developing emotional regulation (Margolin & Gordis, 2000). Living in a disadvantaged neighborhood also exposes the child and family to a host of additional risk factors for early conduct problems and later serious antisocial behavior including fewer high‐quality early childhood education centers and more environmental toxicants (Acevedo‐Garcia et al., 2020). Broader policies at the local, state, and national level shape these neighborhood risk factors (Currie & Gruber, 1996), which are also shaped by history, including histories of structural racism (e.g., redlining, racial covenants; Powell & Porter, 2023) and the politics of where poverty is concentrated (e.g., choices on where to build freeways; Wilson, 2008). Moreover, the intersection of history and policies tend to concentrate multiple risks onto families that have been marginalized in several ways by socioeconomic status, race, and immigration status, among other factors. Risk for conduct problems can also be impacted by policies related to maternal leave (which impacts maternal stress), the availability and cost of high‐quality childcare, birth control access and education (which impacts rates of teen parenthood), and the quality and access to maternal mental health care and prenatal care.

## Nature, nurture, and their interplay

Much of the literature on the coercive cycle and broader context implies that parents and the broader context are socializing agents helping to shape children's behavior via ‘nurture.’ However, is that the case? In most observational studies, children and caregivers share genes and, thus, it is not possible to fully disentangle whether contextual effects are due to the environment.

### Genetics of conduct problems

Twin studies indicate that youth antisocial behavior and child conduct problems are heritable, with ~50% of the variance between youth in antisocial behavior due to genetic factors (Burt, 2022). Adoption studies have also provided evidence for genetic influence, with biological parent antisocial behavior predicting youth antisocial behavior (Burt, 2009b). Heritability varies by the type of behaviors examined, with aggression being more heritable than rule breaking (Burt, 2009a), early onset antisocial behavior more heritable than adolescent onset antisocial behavior (Ferguson, 2010), and antisocial behavior with CU traits more heritable than without CU traits (Viding, Fontaine, & McCrory, 2012). Though the majority of existing twin studies start in middle childhood or later, work in preschoolers indicates substantial heritability of conduct problems and related constructs (Flom & Saudino, 2018). Moreover, during preschool, many of the temperamental risk factors for conduct problems (e.g., attention problems, inhibitory control; Austerberry, Mateen, Fearon, & Ronald, 2022) show moderate to high heritability as well as overlap in heritability with conduct problems. There is also evidence that genes influence the *stability* of conduct problems over time but also that new genetic influences emerge across development. For example, in a longitudinal study from age 4 to 16 years, genetic factors present at age 4 also predicted variance in conduct problems at age 16, with new genetic factors emerging over time (Pingault, Rijsdijk, Zheng, Plomin, & Viding, 2015). Thus, there are clear genetic influences on conduct problems and later forms of antisocial behavior, likely with some stability and change of genes that predict the onset, maintenance, and escalation (or desistence) from conduct problems.

### Genetic influences on parenting

**Figure 3:**
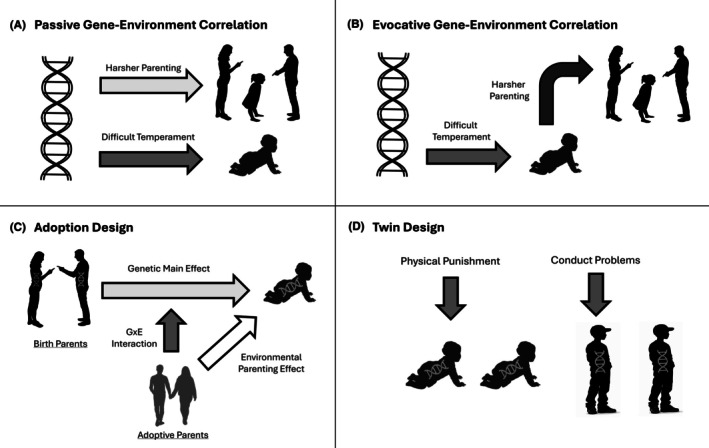
Gene environment interplay and genetically informed designs. (A) passive gene–environment correlation, (B) evocative gene–environment correlation, (C) an adoption design showing genetic, environmental, and G × E interaction effects, (D) twin difference design, showing a nonshared environmental effect of physical punishment

Given the moderate heritability of conduct problems, links between harsh parenting and conduct problems may not reflect environmental effects of parents on children, but could instead reflect shared genes. That is, genes that increase risk for children to engage in problem behaviors may also lead parents to be harsher, meaning that it is not parenting, but rather shared genes, creating the link between harsh parenting and child conduct problems, a process termed *passive gene–environment correlation* (passive rGE). Similarly, children with risky genes may behave in ways that evoke harsh parenting. In this scenario, termed *evocative gene–environment correlation* (evocative rGE), the child's genes, not parenting, may be leading to the correlation between harsh parenting and child conduct problems (see Figure 3).

In fact, several studies support the notion that some of the link between parenting and child conduct problems (and later antisocial behavior), is due to rGE (Burt, 2022; Jaffee, Strait, & Odgers, 2012). For example, in a meta‐analysis of both twin and adoption studies, Klahr and Burt (2014) found that child genetic influences account for over 40% of the variation in harsh parenting the child received during childhood. Interestingly, research from a prospective adoption study, the Early Growth and Development Study (EGDS), has found that evocative effects appear to be activated via child behaviors like anger and negativity. The EGDS is a cohort of >500 linked families with data from biological and adoptive parents and children who were adopted, on average, only a few days after birth (Reiss et al., 2022). In the case of child evocative effects, an analysis of EGDS found that birth parent traits (behavioral activation) were related to the child's anger at age 4.5 years (evidence of a genetic pathway), which in turn was related to adoptive parent's hostility toward the child when the child was 6 years old (evidence of evocative rGE), which was then related to more externalizing problems at age 7 (Shewark et al., 2021). Thus, there is clear evidence that rGE accounts for some of the association between parenting and child behavior, such that this association is not entirely “environmental” and is likely due, at least in part, to genetic factors, some of which are driven by evocative effects of the child's behavior on the parent.

### Environmental effects of parenting

At the same time, genetically informed studies have shown that parenting also has effects that are environmentally mediated (see Figure 3C and 3D). First, studies have examined differences in parenting and child behavior in families with identical twins to test for environmental effects. In this approach, if one twin receives harsher parenting and then goes on to have more conduct problems, the fact that identical twins have the same DNA increases confidence that environmental factors (e.g., parenting) are driving these associations. For example, Burt, Clark, Gershoff, Klump, and Hyde (2021) found that the twin who received harsher parenting was the twin with higher conduct problems during middle childhood, even among identical twins. In the same sample, Waller et al. (2018) found that differences in harsh parenting received between identical twins predicted twin differences in both aggression and CU traits, whereas differences in parental (lower) warmth only predicted twin differences in CU traits.

A second, overlapping approach, is to use twin samples to examine the extent to which the correlation between parenting and behavior problems is genetic versus environmental in origin, using a model that extends the typical ‘ACE’ model to two variables. For example, Tomlinson, Hyde, Dotterer, Klump, and Burt (2022) found that the association between harsh parenting and CU traits during middle childhood was almost entirely due to genetic factors (i.e., gene–environment correlation). However, the association between warmth and CU traits was partially genetically and partially environmentally (~50% each) mediated, which provides additional evidence that this pathway has both rGE and environmentally mediated effects.

A third approach has been to examine children who have been adopted, which eliminates passive rGE when studying the postnatal rearing environment. For example, using the EGDS cohort, one study found that adoptive mothers' and adoptive fathers' hostile parenting predicted toddlers' aggressive behavior when children were 27 months old (Stover et al., 2012). In another example in the EGDS, Hyde et al. (2016) found that birth parent history of antisocial behavior predicted CU behaviors at 27 months (evidence of genetic effects), but also that adoptive parent positive reinforcement predicted CU behaviors (evidence of an environmental pathway). In this same cohort, Trentacosta et al. (2019) found that parental harshness and CU behaviors predicted each other reciprocally from 27 to 54 months. These findings show evidence both that CU behaviors may evoke harsher parenting (even from a parent who does not share genes with the child) and that harsh parenting does have an impact on CU behaviors, over and above these child effects. Interestingly, in both of these studies, there was also evidence that genetic risk interacted with these pathways, highlighting the potential for gene × environment interactions (G × E) that are described in the next section. However, before turning to G × E interactions, it is important to note that adoption designs do not eliminate passive rGE for prenatal exposures like maternal smoking during pregnancy because in a standard adoption design the same person (the birth mother) is providing both genes and the prenatal environment. Interestingly, a review of studies without this confound (e.g., IVF designs) found that there was no evidence for a causal association between maternal smoking during pregnancy and offspring conduct problems (Rice et al., 2018).

### Gene × environment interactions

Studies suggest that environmentally mediated effects are sometimes further qualified by G × E interactions (see Figure 3C). Though there was initial hope to use molecular genetics to test G × E interactions with specific measured genes (e.g., Byrd & Manuck, 2014), much of the most compelling evidence for G × E interactions comes from twin and adoption studies. For example, in several of the examples described above, there were evidence of G × E interactions in addition to main effects of parenting. In work examining CU behaviors in the EGDS, Hyde et al. (2016) not only found evidence for genetic (birth parent effects) and environmental (adoptive parent effects) influences, but also an interaction. In this case, though greater birth mother history of antisocial behavior predicted child CU behaviors at age 27 months (genetic effect), if these children had parents who showed high levels of positive reinforcement during an observational toy clean up task at age 18 months, then the association between birth parent and child effects was diminished. That is, children's genetic risk only translated into CU traits when their adoptive parents were lower on positive parenting, which provides evidence that adoptive parents' positive parenting can ‘knock out’ these genetic effects, at least during this early developmental period. In very similar findings in middle childhood using a twin design, Tomlinson et al. (2022) found that CU traits were moderately heritable, but that the heritability of CU traits decreased when parents were high on involvement and warmth and low on harshness.

G × E interactions are not limited to the parent–child relationship and may extend to parental factors like parental mental health as well as many of the broader risk factors described in ‘The Broader Context’ section. For example, in the EGDS, adoptive mother mental health moderated the association between genetic risk and an early precursor to conduct problems (infant attention to frustrating events), such that genetic risk was only related to infant attention to frustrating events when adoptive mothers reported more symptoms of anxiety and depression (Leve et al., 2010). In another EGDS study that focused on an important aspect of the broader context in early childhood, attending center‐based early childcare was associated with increasing conduct problems across early childhood, but only when there was also genetic risk for dysregulation (Lipscomb et al., 2014).

A key challenge with examining G × E interactions involving broader contextual risk factors is that these factors are difficult to study using genetically informed designs (e.g., there may not be much variability among adoptive parents in socioeconomic status). However, there are several examples of G × E interactions in the literature related to broader context. For example, several studies in adolescence have shown that socioeconomic status and deviant peer affiliation moderate genetic risk (Burt, 2022). Regarding the socioeconomic context, antisocial behavior appears to be less heritable in contexts with fewer resources (e.g., lower family income, living in a lower income neighborhood). This pattern of findings is consistent with a bioecological model or a ‘social push’ hypothesis in which in more disadvantaged contexts there is more ‘push’ through more risk opportunities (e.g., greater rewards for antisocial behavior, more models of this behavior in the neighborhood), leading more youth to engage in antisocial behavior, even in the absence of genetic risk (Burt, 2022; Hyde, Tillem, Westerman, & Guzman, 2024). Because most of these studies have focused on adolescence or late childhood, we know less about how this gene–environment interplay may occur in broader contexts during the toddler and preschool years, particularly since this is a period when children are spending more time with their parents and less time in their neighborhood.

G × E interactions may be another way of measuring *goodness of fit* between parenting and child temperament as described above. In addition, G × E interactions may also reflect *differential susceptibility* (Belsky & Pluess, 2009), a theory that posits that some youth are more susceptible to both positive and negative environments. For example, in a sample of 1–3‐year‐old children, those with difficult temperaments were more susceptible to harsh parenting (i.e., they showed more conduct problems) and to supportive parenting (i.e., they showed fewer conduct problems), compared with children with relatively easy temperaments (Van Zeijl et al., 2007). In an example with a measured genotype and in the context of a parenting intervention described below, the effects of the Family Check‐Up were moderated by genetic risk, an example of a gene by intervention (G × I) interaction (Shaw et al., 2019). In this case, for youth with low genetic risk (as measured by a polygenic risk score), assignment to the intervention (vs. control) group did not predict the trajectory of conduct problems from age 2 to age 14. However, for those high on genetic risk, the intervention predicted a higher likelihood of being persistently low in conduct problems, whereas membership in the control group predicted a higher likelihood of being persistently high in conduct problems.

### Conclusion: Nature and nurture

There are several important takeaways from the genetically informed studies: First, there is substantial evidence that some of the effects of context (e.g., parenting) on conduct problems that have been found in observational studies are actually genetically mediated. Second, there is robust evidence for child effects on parenting that are at least partially genetically mediated. That is, even when parents are not genetically related to their children, their children's behavior and temperament, which is influenced by genetics, evokes different parenting behaviors. This observation is consistent with the coercive cycles we have described above – that is, child effects can influence parenting (Bell, 1968). Third, there is evidence across multiple types of studies (e.g., adoption, twin) that parenting does have some of its effect through environmentally mediated (i.e., ‘nurture’) pathways. Fourth, there are clear transactions over time in which child effects impact parenting, which, in turn, impacts child behavior. These observations are consistent with the unfolding of coercive cycles over time in which parent and child behaviors cycle across development, and these genetically informed designs help to show the genetic and environmental mechanisms through which these cycles occur. Fifth, there is evidence for multiple forms of GxE interactions. In one key pattern of GxE interaction, positive parenting mitigates genetic risk, which is important information for parents to know – that even with a more difficult child with genetic risk for conduct problems, if parents are able to shift to more positive parenting approaches, they may be able to dampen the impact of genetic risk, which strengthens the rationale for early interventions. Sixth, G × I interactions may also exist, meaning that interventions may have more or less impact depending on child genetics, highlighting the need for personalized interventions. Seventh, it is important to note that most families consist of parents and children who share genes, and thus prevention and intervention must contend with the challenge that parents and children may share similar risk factors (e.g., impulsivity, anger proneness) and that these risk factors are embedded within contexts that may make shifting parent–child interaction cycles more challenging. Eighth, one type of rGE we have not described in detail is the idea that individuals choose or seek out their environments, which is termed active rGE. Though toddlers and preschoolers have less ability to seek out specific environments, as children develop, they have more opportunities to seek out experiences with specific peer groups or risky activities like experimenting with substance use. Thus, later in development, genes plus environments can play a role in the ways in which conduct problems escalate and become more chronic because youth may seek riskier environments that match their interests. This observation has major implications for the need for early interventions to prevent these cycles that may expand in scope and, thus, become less malleable over time (Baskin‐Sommers et al., 2024).

Finally, beyond parenting and parent–child effects, it is also important to consider whether the broader set of contextual risk factors we have laid out above are actually ‘environmental’ in origin (see Burt, 2022; Jaffee et al., 2012 for a review of this question). Many of these factors cannot be tested with genetically informed or experimental models. However, a variety of quasi‐experimental studies have shown that several of these risk factors (e.g., neighborhood disadvantage, maternal depression) do seem to have a causal environmental influence (Jaffee et al., 2012). However, effect sizes for many risk factors in the development of early conduct problems and later antisocial behavior are inflated when relying on evidence from observational studies (Rice et al., 2018), which further underscores the importance of quasi‐experimental designs (Duncan, Magnuson, & Ludwig, 2004).

Having laid out the processes by which parents, children, and the broader context can ‘set the stage’ for early conduct problems and coercive cycles that can ultimately lead conduct problems to persist and escalate, the next question we ask is: What are the key mechanisms that link these risky genetic and environmental factors to conduct problems? We turn to the brain to examine the neural correlates of antisocial behavior in youth and the potential to identify early neural markers of risk for conduct problems in young children.

## Brain mechanisms

### Neural correlates

At the broadest level, youth antisocial behavior has been theorized to be underpinned by differences in the structure and function of three key circuits: (1) The cortico‐limbic circuit, with the amygdala as its hub, which is key for emotion, emotion regulation, threat detection, fear, and learning contingencies, among other functions; (2) The cortico‐striatal circuit, with the ventral striatum as a key hub, which is central for reward processing and motivation; and (3) The prefrontal cortex, which is key for inhibition, working memory, and other aspects of executive function and cognitive control (Blair, Leibenluft, & Pine, 2014; Hyde et al., 2024; Viding & McCrory, 2012). These circuits are hypothesized to be central to understanding antisocial behavior because they are linked to the neurocognitive processes and behaviors disrupted in youth who engage in high levels of antisocial behavior (Hyde, Shaw, & Hariri, 2013). That is, these youth tend to show difficulty with emotion regulation (Toro, García‐García, & Zaldívar‐Basurto, 2020) and with recognizing and processing emotion in others (Marsh & Blair, 2008; Trentacosta & Fine, 2010), they perseverate on behaviors that were previously rewarded but are now being punished and tend to over emphasize approach over inhibition (Byrd, Loeber, & Pardini, 2013), and they have difficulty inhibiting problematic behaviors (Vize, Miller, & Lynam, 2018). Moreover, individual differences in these brain circuits also connect to the temperamental risk factors for conduct problems that we have discussed previously. For example, deficits in amygdala function are hypothesized to contribute to fearlessness, lack of prefrontal control, and excessive reward can lead to challenges in inhibitory control, which, along with emotion dysregulation, can lead to challenges in self‐regulation and effortful control (Hyde, Shaw, & Hariri, 2013).

Most theory in this area hypothesizes that individual differences in these brain circuits emerge very early in life. However, most empirical literature in this area focuses on adolescents. At this later developmental period, meta‐analyses (Rogers & De Brito, 2016) and mega‐analyses (Gao et al., 2024) of structural brain imaging, as well as task‐based functional MRI (Alegria, Radua, & Rubia, 2016), mostly support these theories in identifying consistent differences in these specific brain networks. However, much of this work also finds that conduct problems are associated with differences in structure and function more broadly/diffusely across the brain (for more see Hyde et al., 2024). Similar patterns of diffuse neural differences associated with youth antisocial behavior are seen with diffusion imaging measuring white matter tracts (Waller, Dotterer, Murray, Maxwell, & Hyde, 2017), and using network neuroscience to look at large scale functional brain networks (Dugré & Potvin, 2021).

### Risk factor to brain connections

Interestingly, in a separate and growing literature, developmental neuroscientists are beginning to connect exposure to various adversities that are also linked to conduct problems to many of the same regions of interest just highlighted, including the amygdala, ventral striatum, and areas of the prefrontal cortex. For example, harsh parenting has been connected in multiple studies to the structure and functioning of the amygdala (Farber, Gee, & Hariri, 2022; Gard et al., 2022). In fact, in a sample of at‐risk boys, observations of harsh parenting at age 2 predicted differences in amygdala reactivity at age 20, which in turn predicted antisocial behavior (Gard et al., 2017). This type of study suggests that one mechanism through which risk factors like harsh parenting increase risk for youth antisocial behavior is via their impact on the developing brain. In addition to studies, linking harsh parenting to amygdala structure and function, there are also studies linking other contextual risk factors like neighborhood disadvantage (Suarez, Burt, Gard, Klump, & Hyde, 2024), family poverty (Johnson, Riis, & Noble, 2016), and exposure to community violence (Suarez et al., 2024) to activity and structure in key regions like the amygdala (Suarez et al., 2024), ventral striatum (Westerman et al., 2024), and prefrontal cortex (Tomlinson et al., 2020). This growing literature suggests that understanding these brain mechanisms may help elucidate the biological embedding of these experiences (for reviews, see Hyde, Gard, Tomlinson, Suarez, & Westerman, 2022; Johnson et al., 2016) and also help us understand how and why these specific experiences increase risk for youth antisocial behavior (Hyde et al., 2024).

### Early brain development

Though these emerging models are promising, the existing neuroimaging literature almost exclusively focuses on adolescence because it is challenging to engage in large‐scale neuroimaging with toddlers, preschoolers, and young school‐age children. Though a few studies (e.g., Gard et al., 2022) have used longitudinal data to connect parenting in the toddler and preschool period to later brain function, they are rare. Fortunately, there are now studies using prenatal and infant neuroimaging to connect brain structure and function in this early period to markers of risk for conduct problems.

Brain development is especially rapid in the early years, with the brain's basic structural and functional framework in place by the second year of life, or even earlier (Gilmore, Knickmeyer, & Gao, 2018). Although research on links between infant brain development and early conduct problems and related temperament markers is quite limited, recent findings highlight many of the same regions of interest that were reviewed above. For example, in a study of newborns, larger right amygdala volume and stronger left amygdala connectivity mediated the association between a marker of maternal inflammation during pregnancy and lower impulse control during the toddler years (Graham et al., 2018). In another study, striatum‐frontopolar connectivity during resting fMRI mediated the association between family socioeconomic status and externalizing problems at age 2 (Ramphal et al., 2020). In a study of newborns, stronger functional connectivity between the cingulo‐opercular network (an executive network that exerts top‐down control) and the medial prefrontal cortex in the first months of life was associated with CU behavior at age 3 (Brady et al., 2024). Looking even earlier, follow‐up of a cohort with fetal neuroimaging indicated that weaker in utero functional coupling between two networks – the subcortical limbic network and the medial prefrontal network – was linked with aggression at age 3 years (Hendrix et al., 2023). Finally, other research at this very early period also implicates brain structures and regions that have received less attention in research on antisocial behavior in adolescence. For example, in a multimodal MRI study of the cerebellum among neonates, a specific cerebellar functional gradient cluster was associated with toddlers' externalizing problems (Kim, Kapse, Limperopoulos, & De Asis‐Cruz, 2024).

New studies like the Healthy Brain Cognitive Development (HBCD) study, which aims to recruit over 7,000 mothers and infants across 27 sites, will provide highly valuable neuroimaging data beginning in infancy (Dean III et al., 2024), along with EEG, biospecimens, and assessments of prenatal exposures, family and contextual risk and protective factors, and infant temperament and behaviors, including early conduct problems. We hypothesize that what will be key to understanding early conduct problems will be to identify the ways in which early brain development is on a delayed or different course for youth with early conduct problems or risky temperament characteristics. That is, during the first 5 years of life, there is tremendous brain development that leads to outcomes like walking and talking via greater motor control and development of language processing regions. As we have emphasized, most older infants and toddlers initially show behaviors like temper tantrums, difficulty inhibiting responses, and non‐compliance, but then desist from these behaviors. Their desistance is likely due, in part, to the fact that areas of the brain related to behavioral and emotional regulation (e.g., the circuits described above) develop rapidly to support the tasks of planned action and emotion regulation (Salzwedel et al., 2019). Thus, we expect that individual differences in the development of these brain areas early in life are key to understanding the course of early conduct problems, a notion that will hopefully be tested with these new data sources in the coming decade.

### Summary and next steps in early brain research related to conduct problems

In sum, research at later developmental periods has begun to identify key brain networks that may differ in structure, function, or connectivity in youth engaged in antisocial behavior. These circuits may be shaped by risk contexts for youth antisocial behavior, providing a potential neuro‐mechanistic model to understand how early experiences shape the trajectory of conduct problems into serious antisocial behavior (see Hyde et al., 2024). However, we still have relatively little data prior to adolescence and, thus, do not yet know whether these neural mechanisms will extend to early developing conduct problems, nor what these trajectories look like across development. We hypothesize that early prenatal risk and genetics shape individual differences in the developing brain that yield temperamental risk for early conduct problems (e.g., fearlessness, low effortful control), which, in turn, makes parenting more challenging. These early parenting interchanges, along with broader contextual influences (e.g., toxicant exposures), continue to shape brain development in ways that lead to greater engagement in problem behavior and difficulty with inhibition and emotion regulation. Thus, the brain itself becomes a mechanism in these coercive cycles (Baskin‐Sommers et al., 2024).

## Biopsychosocial cascades

**Figure 4:**
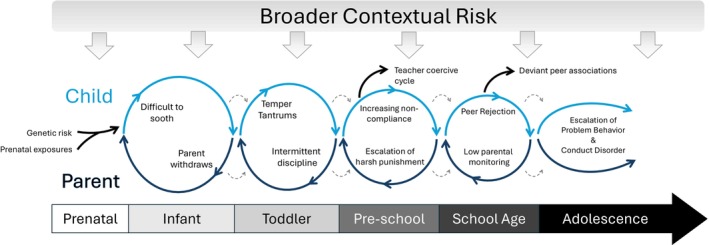
An unfolding of risk for conduct problems over time in the parent–child relationship. A Developmental Timeline: Risk for child conduct problems may start at conception and is built on generational and structural histories that can yield, as one key example, young parents who face structural disadvantage and low social support in a society that has few evidence‐based policies to support parents of young children. During gestation, the developing fetus may encounter prenatal exposures that exacerbate existing genetic risk. After birth, the child may be more difficult to soothe with greater emotion (e.g., crying) and a parent who is depressed and without the financial or emotional skills to engage warmly with a more difficult infant. These processes reflect early GxE interactions. As the child begins crawling and then toddling, their emerging mobility co‐occurs with rapid brain development, particularly in ‘bottom up’ circuits that drive emotion and motivation toward reward (without the same pace of improvements in neurobehavioral control). Normatively, this yields a toddler who can get into new, risky situations with clear desires, leading to temper tantrums and the emergence of problematic behavior. This toddler may also be lagging in some aspects of brain development, leading to greater temper tantrums, slower development of speech, and difficulty expressing emotions. These rapid changes, along with a more difficult temperament (e.g., fearlessness, low effortful control, reward sensitivity), are a major challenge to a parent who may already be struggling to bond with the child and manage their own emotions. During this period and into the preschool years, the coercive cycle emerges, where the child learns through many micro‐exchanges that tantrums and non‐compliance eventually work to achieve their goals, and the parent is reinforced for giving in and sometimes reinforced for harsh parenting, resulting in inconsistent and harsh parenting that further undermines the parent–child relationship. This behavioral cycle may also be a manifestation of genes the parent and child share (e.g., for poor emotion regulation), and an interaction (e.g., harsh parenting exacerbating the child's genetic risk). These experiences and individual differences in brain development yield a different trajectory of brain development in circuits supporting fear, learning, emotion regulation, behavioral control, and reward seeking. These coercive cycles can then expand to teachers in childcare and preschool (and eventually elementary school), disrupting relationships and opportunities for the child to learn more prosocial behavior and disrupting schooling, potentially leading to school failure. These processes can then lead to disruptions in peer relationships (rejection by normative peers, acceptance by deviant peers), which may be exacerbated by living in areas with disadvantaged schools and communities that have social ties undermined by concentrated disadvantage, all processes shaped by local and national policies, as well as history. As development proceeds, these cycles may become more entrenched as they shape the contexts available to the child and as the child seeks out specific environments, making it more challenging to intervene

From early prenatal and genetic risk to dyadic cycles to broader contextual influences filtering through brain and behavioral development, the development of early conduct problems is complex across multiple levels (See Figure 1) and timescales (see Figure 4 for the unfolding of this risk and a developmental timeline). As development proceeds, these cycles may become more entrenched as they shape the contexts available to the child and as the child seeks out specific environments, making it more challenging to intervene. However, through intervention or through additional supports for parents (or other caregivers becoming engaged), these cycles can be disrupted to yield new cycles that include positive child behavior and positive parenting skills. These pathways are certainly not destiny, with ample desistence and resilience even among children and families with high risk. Moreover, as emphasized throughout, there are tremendous individual differences at each step, with equifinality and multifinality in pathways, yielding a tremendous number of different pathways to and away from early conduct problems throughout development.

## Intervention and prevention

### Intervention

Evidence‐based interventions for addressing conduct problems among young children typically engage parents as the main agents of change for shaping children's behaviors. In a recent review of evidence‐based psychosocial treatments for childhood disruptive behaviors, all of the treatments meeting the highest well‐established level involved parents (Kaminski, Claussen, Sims, & Bhupalam, 2024). Well‐established treatments include group parent behavior therapy such as the Incredible Years (Webster‐Stratton & Reid, 2018), parent‐focused behavior therapy such as Generation Parent Management Training‐The Oregon Model (PMTO) (formerly Parent Management Training – Oregon Model; Forgatch & Gewirtz, 2017) and parent behavior therapy that includes children such as Parent–Child Interaction Therapy (PCIT; Zisser‐Nathenson, Herschell, & Eyberg, 2017). These evidence‐based parent‐training programs are deeply rooted in the empirical literature focused on parent–child coercive cycles and seek to change these cycles from negative to more positive. They use social learning theories of behavior problems to teach parents behavioral parenting skills including positive disciplinary strategies and work to increase positive parenting (e.g., positive attention for positive child behavior). Thus, they are a strong example of how long‐standing social developmental research has informed a host of effective interventions.

Many of these programs target preschool or young school‐aged children, with recent adaptations targeting infants and young toddlers to treat and prevent the earliest emerging conduct problems. One such program, Parent–Child Interaction Therapy‐Toddler (PCIT‐T; Kohlhoff et al., 2024) is an adaptation of PCIT. PCIT‐T adds a stronger emphasis on the parent–child attachment relationship relative to PCIT and focuses on parental sensitivity as a more explicit target of intervention, with recent evidence that this intervention is superior to other interventions and waitlist controls (Kohlhoff et al., 2024). Another recent adaptation of PCIT focuses on preschoolers with CU traits, with an initial pilot study providing evidence of decreases in conduct problems and CU traits as well as increases in empathy (Kimonis et al., 2019).

### Prevention

A substantial number of trials, starting decades ago, established the efficacy of programs to prevent child conduct problems, though many of the earliest trials targeted conduct problems during the elementary school years. For example, Fast Track was a large multi‐site trial that began in the 1990s by providing a comprehensive set of family‐ and school‐based programming to families throughout the elementary school years (Bierman et al., 2019), with robust evidence of positive impacts of the intervention into adulthood, including reductions in psychopathology and criminal convictions and improvements in well‐being. In addition, there is an even longer history of broader interventions targeting environments like high‐quality preschool resources that were aimed at promoting academic success and have shown important effects on preventing later antisocial behavior (e.g., Heckman & Karapakula, 2019; Reynolds, Ou, Mondi, & Giovanelli, 2019).

In more recent decades, there has been an increasing emphasis on specifically preventing early conduct problems before the elementary school years, beginning during the toddler period. For example, the Family Check‐up (FCU) for toddlers is a family‐centered prevention program that involves an assessment of the child and the family, an initial ‘get‐to‐know‐you’ meeting with the family, and a formal feedback session about strengths and risks in the family and broader context, guided by the empirical literature we have summarized. At the feedback session, families are offered follow‐up services including empirically supported interventions that are tailored to the family's unique constellation of risks and needs. One defining feature of this approach is that it can include parent management training, but it can also address issues in the broader context that have been linked to the development of conduct problems (e.g., parent depression). This program has been supported in multiple randomized trials showing reductions in problem behaviors via parental involvement and positive parenting (Dishion et al., 2008; Shaw, Dishion, Supplee, Gardner, & Arnds, 2006), as well as other risk factors (e.g., maternal depression; Shaw, Connell, Dishion, Wilson, & Gardner, 2009), with positive effects into adolescence via improvements in child inhibitory control (Hentges et al., 2020).

The FCU has now been integrated into tiered models, including the Smart Beginnings project and the Pittsburgh Study. In Smart Beginnings, the FCU is considered a secondary prevention model while paired with the Video Interaction Project (VIP) model, which is a universal primary prevention model targeting positive parenting, with implementation across the early years of life in primary care. A recent evaluation of Smart Beginnings indicates that involvement in this tiered preventive intervention led to reductions in early childhood conduct problems via reductions in negative affect during discipline (Shaw, Mendelsohn, Morris‐Perez, & Krug, 2024). The Pittsburgh Study is another tiered model that includes an even broader set of platforms beyond VIP and the FCU, with implementation both in‐person and remotely across health care and various other community settings. Initial findings suggest high levels of enrollment in parenting programs, with parents experiencing higher levels of adversity being especially likely to opt into one of the parenting programs (Krug, Mendelsohn, Wuerth, Roby, & Shaw, 2025). These tiered approaches are viewed as an essential public health strategy to promote early relational health, which can buffer risk for early conduct problems.

Prevention can begin even before the toddler years, and the Nurse–Family Partnership (NFP) is one key example of the potential of early prevention. In the NFP, nurses provide home visits to low‐income or at‐risk families during pregnancy and infancy, with overarching goals to improve the mother's well‐being and parental caretaking. Although the NFP was not specifically designed to prevent early conduct problems, its focus on maternal well‐being and caregiving can have downstream effects on child behavior, with initial longitudinal follow‐ups finding that participation in the NFP reduced adolescent offspring's engagement in specific antisocial behaviors (Olds et al., 1998; though see Kitzman et al., 2019). These early‐life approaches have continued to evolve in recent decades. For example, the Family Connects intervention also involves nurse home visiting, with initial evidence supporting high‐quality implementation, reductions in emergency medical care, and fewer investigations for suspected child maltreatment (Goodman, Dodge, Bai, Murphy, & O'Donnell, 2021).

Overall, themes that have emerged in recent prevention research and that we see as critical to increasing the effectiveness of prevention and intervention, include (1) starting earlier in life (e.g., with nurse visitation or early parenting in the toddler years); (2) providing personalization through flexible treatment menus that target the myriad of risk factors for child conduct problems; (3) tiered models in which families may get brief, ‘light touch’ early interventions, with the ability to provide more intensive services as needed based on the response to initial attempts; (4) the idea of ‘check‐ups’ in accessible locations – that is embedding services within existing services for families (e.g., Women, Infant, Child Centers; primary care) with regular (e.g., yearly) check‐ins on child behavior; (5) targeting preventive interventions to families based on the basic science of which risk factors increase the potential need for service; and (6) adding components to engage families such as motivational enhancements or feedback on parent and child behavior (Dodge, 2019; Shaw et al., 2024). Next steps in the field include building innovative interventions using dismantling studies, just in time interventions (Klasnja et al., 2015), and Sequential Multiple Assignment Randomized Trials (SMARTs) that can examine whether specific sequences of interventions increase their impact and/or how treatments can be tailored based on child/family progress during treatment (Kidwell & Hyde, 2016). Moreover, trials that test mechanisms, like brain development, can both inform the basic science of the development of early conduct problems and elucidate mechanisms of intervention (Valadez, Tottenham, Tabachnick, & Dozier, 2020). Finally, additional steps are needed in the policy and implementation realm in terms of making intervention and prevention accessible and broadly available.

## Future directions and research needs in the area

Research into the development of youth antisocial behavior has given rise to a host of effective treatments. However, though parent management‐related interventions are effective, they are not effective for all youth (Lundahl, Risser, & Lovejoy, 2006), they may not currently yield a large enough effect to bring some youth with some of the most severe behavior (e.g., those with CU behaviors) into a normative range of behavior (Perlstein, Fair, Hong, & Waller, 2023), many families cannot access or pay for these evidence‐based services (McGoron & Ondersma, 2015), and some studies suggest that the effects of intervention may not be long lasting (Lundahl et al., 2006). To address these challenges, leading prevention and interventionists in the field have been offering some important next steps for the field that were outlined above (Dodge, 2019; Shaw et al., 2024). However, beyond these intervention‐focused directions, there are also important new basic to translational directions needed in the field:

First, conduct problems need to be viewed and treated more broadly as a neurodevelopmental disorder. Like other neurodevelopmental disorders (e.g., autism, ADHD), conduct problems and later antisocial behavior have their origins in early childhood, are characterized by disruptions in brain and neurocognitive development, have significant genetic influences, and, as described above, persist throughout development for some youth (often into adulthood) with impaired functioning across multiple domains (e.g., social, educational, professional; Van Goozen, Langley, & Hobson, 2022). Yet, conduct problems are often seen simply as ‘bad behavior’, rather than a mental health challenge in need of support, including widely accessible early screening and intervention. On the other hand, parents may often take a ‘wait and see’ approach, perhaps because they believe that their children's behaviors are only ‘a phase’ (McGoron & Ondersma, 2015). Perhaps both of these issues are due to the stigmatization of conduct problems and related disorders, with fewer advocates than other neurodevelopmental disorders (e.g., autism), potentially because those affected by conduct problems (e.g., parents, youth) worry about stigma from these disorders and because the risk factors for conduct problems (e.g., disadvantage) mean that many of those affected do not have the resources, nor power, to advocate. Building understanding and compassion for children with conduct problems and youth with later antisocial behavior does not imply condoning or supporting these negative behaviors, nor does it eradicate the need for accountability. In contrast, greater compassion could push our society to see conduct problems as a public health concern to be addressed with science, public health campaigns, prevention, and effective intervention, rather than focusing only on punishment once behavior has escalated (Baskin‐Sommers, Ruiz, Sarcos, & Simmons, 2022).

Second, one challenge in understanding the basic science of the development of conduct problems in early childhood and beyond is one of measurement. That is, there is substantial heterotypic continuity in the behaviors (i.e., the same underlying behavioral challenges look different at different ages and stages; e.g., Loeber & Hay, 1997) involved in the escalation from conduct problems to antisocial behavior (Petersen, 2024). For example, the same child with persistent conduct problems may show substantial problems with temper tantrums during early child, which may transition to proactive aggression in adolescence. These types of changing presentations lead to challenges in measuring continuity and change. Promising approaches to address these challenges include better understanding the general and specific neurocognitive correlates as they change across development (Hyde, 2015; Nigg, 2017) and leveraging integrative data techniques to better understand developmental scaling (Petersen, Choe, & LeBeau, 2020).

Third, one key question for efficient prevention approaches is to better understand how early we can identify youth and families at risk to engage in preventative interventions. The balance in this issue is between effective and efficient prevention before coercive cycles begin/become entrenched and the potential issues of false positives, self‐fulfilling prophecies, and stigmatizing youth and families. For example, some research is beginning to identify markers of CU traits in the toddler period, and though there is thoughtful work emphasizing that CU behaviors in this period are only a *risk factor* for CU traits, which itself is only a risk factor for later psychopathy, one can worry that this research can be misconstrued to be studying ‘preschool psychopaths’, a misreading that could have major negative consequences (Waller & Hyde, 2017). Thus, scientists and practitioners have an important role in educating the public about risk factors and prevention versus stability and destiny. To be clear, none of the work we review here should be construed to demonstrate that any child with early conduct problems is destined to show serious and chronic antisocial behavior. At the same time, we see work examining issues like ‘when to worry’ that can be embedded within primary care or other more universal settings as having great potential for helping to identify who will and who will not desist and, therefore, who would be best targeted through preventive interventions (Wakschlag et al., 2019).

Fourth, though the field is already moving in this direction, it is clear that to have a higher rate of success with prevention and intervention, we need to better identify who is at most risk to target for prevention, then we need ways to personalize these interventions to these youth and families, and then make these interventions accessible and easy to engage in, integrated into regular healthcare so that they can be utilized across development, as well as scaled in ways that are sustainable, including through motivational enhancements and technological innovations (McGoron et al., 2024; McGoron & Ondersma, 2015). Moreover, increasing evidence suggests that targeting many of the risk factors in the caregiving environment reviewed in this paper (Shelleby & Shaw, 2014), such as parental emotion regulation (Byrd, Lee, Frigoletto, Zalewski, & Stepp, 2021; Hajal & Paley, 2020), mental representations (Julian et al., 2018), and psychopathology (Shaw et al., 2009; Zalewski, Lewis, & Martin, 2018), may help to augment existing parenting interventions (Camoirano, 2017; Maliken & Katz, 2013). Better targeting the broader risk context, beyond just parenting, appears to be key to recent interventions (e.g., the Family Check‐Up) and may help make interventions more effective for more families. Ultimately, for these efforts to be effective, families need to be able to access (and afford) evidence‐based care.

Fifth, although the parent–child dyad may be a proximal mechanism for understanding conduct problems, this dyad is embedded within broader systems and cultural contexts. From a public health perspective, preventing the conditions that promote conduct problems may be more effective in the early years of life and place less onus on parents and families. For example, programs like those that move families to a less disadvantaged neighborhood or provide low‐cost, high‐quality preschool programs may be more effective and have broader public health impacts. More research is needed into broader structural approaches that start early in life (or before birth) with longer‐term follow‐ups. We should also not lose sight of the fact that there is considerable diversity in parenting and conduct problems, both within and across cultures around the globe (Lansford, 2022). Thus, there is no ‘one‐size‐fits‐all’ method to working with parents to address and prevent early conduct problems, and it is incumbent on researchers and policy makers to develop and implement culturally informed approaches.

## Ethics statement

No human subjects data were accessed or analyzed for this report and thus IRB approval and informed consent was not included.

**Key points**

**What is known?**

- Conduct problems emerge from coercive cycles between caregivers and children.

**What is new?**

- Genetically informed research highlights genetic, environmental, and gene × environment interaction interplay that shape risk for conduct problems.
- Adversities that increase risk for conduct problems also impact brain development, highlighting a potential neuromechanistic pathway.

**What is relevant?**

- Advances in understanding the causes of conduct problems are informing new interventions that are personalized and impact multiple risk factors.
- Understanding the nature and nurture of conduct problems can inform public policy to better prevent the ecologies that promote risk.

## Data Availability

Data sharing is not applicable to this article as no new data were created or analyzed in this study.
